# Coronavirus disease-19 and headache; impact on pre-existing and characteristics of de novo: a cross-sectional study

**DOI:** 10.1186/s10194-021-01314-7

**Published:** 2021-08-21

**Authors:** Jasem Youssef Al-Hashel, Fathi Abokalawa, Maram Alenzi, Raed Alroughani, Samar Farouk Ahmed

**Affiliations:** 1 Neurology Department, Ibn Sina Hospital, P.O. Box 25427, 13115 Safat, Kuwait; 2 Faculty of Medicine, Kuwait University, P.O. Box 24923, 13110 Safat, Kuwait; 3 Internal Medicine Department, Farwaniyah Hospital, Kuwait city, Kuwait; 4 Division of Neurology, Amiri Hospital, Arabian Gulf Street, 13041 Sharq, Kuwait; 5 Neuropsychiatry department, Faculty of Medicine, Al-Minia University, P.O. Box 61519, Minia City, 61111 Egypt

**Keywords:** COVID-19, Headache disorders, migraine headache, De novo headache

## Abstract

**Background:**

Coronavirus disease-19 is caused by the severe acute respiratory syndrome coronavirus 2 Headache is a common symptom during and after Coronavirus disease-19. We aimed to study headache character in relation to COVID-19.

**Methods:**

This was a cross-sectional study. Patients who had Coronavirus disease-19, confirmed by reverse transcription polymerase chain reaction technique and presented to the headache clinic within 3 months after the onset of infections were identified to the study. Study included patients diagnosed as primary headache disorders according to The International Classification of Headache Disorders, 3rd edition. Participants were grouped into categories according to having previous or de novo headache. Descriptive data, paired sample *t*-test and the chi-squared test (*X*^2^) were used for statistical analyses of the data.

**Results:**

A total of 121 patients were included in this study. Their mean age was 35.29 + 9.54 and most of them were females (83.5%). Prior to Coronavirus disease-19 infections, 78 (64.5%) had migraine and 11(9.1%) experienced a tension-type headache while 32 (26.4) reported de novo headache post Coronavirus disease-19. Patient had significant increase in headache days 11.09 ± 8.45 post Coronavirus disease-19 compared with 8.66 ± 7.49 headache days before Coronavirus disease-19 infection (*p* < 0.006). Post Coronavirus disease-19, the usage of analgesic increased significantly by the patient with migraine (2.31 ± 1.65 vs 3.05 ± 2.09, *p* = 0.002) while the patient with tension type headache had statistically significant increase in severity (5.556 ± 1.86 vs 7 ± 2.25, *p* = 0.033) and frequency (7 ± 6.29 vs 12.72 ± 7.96, *p* = 0.006) of headache attacks. Bi-frontal and temporal headache are the most reported (40.6% each) headache site among de novo headache group. Patients younger than 40 years had longer duration of the headache attack (18.50 ± 16.44 vs 5.5 ± 9.07, *p* = 0.045) post COVID-19. Male patients compared to females (8.66 ± 1.15 versus 5.93 ± 2.01 *p* = 0.04) had more severe headache post Coronavirus disease-19. De novo headache resolved within 1 month in most of patients (65.3%).

**Conclusion:**

Primary headache get worse after Coronavirus disease-19. De novo primary headache is frequent post Coronavirus disease-19 and resolve within 1 month. Headaches related to Coronavirus disease-19 are severe, present as migraine phenotype. Young male patients with Coronavirus disease-19 tend to have worse headache.

## Introduction

Coronavirus disease-19 (COVID-19) is caused by the severe acute respiratory syndrome coronavirus 2 (SARS-COV-2) that first emerged in Wuhan by the end of 2019 [1].

Patients with SARS-CoV-2 infection can experience a wide range of clinical manifestations, from no symptoms to critical illness. Approximately 33% of people with SARS-CoV-2 infection never develop symptoms [2]. The most common clinical symptoms of COVID-19 infection involve fever, cough, myalgia, and fatigue [3].

However, various neurologic manifestations such as headache, dizziness, anosmia, impaired consciousness, and acute cerebrovascular disease have been also reported [4]. Although Headache was included by the Centre for Disease Control and Prevention [5] as one of the main symptoms of COVID-19, a better definition of COVID-19 related headache and its characteristics are lacking and no definite data on its evolution are available at present.

Headache attributed to systemic viral infection is included in the International Classification of Headache Disorders third edition (ICHD-3) [6] and, although commonly reported [7], specific data are lacking.

It is estimated that with the COVID-19 pandemic there has been a five-fold increase in the incidence of headache in the affected regions [8]. The prevalence of headache was calculated at 10.9% (8.6–13.5%) in a meta-analysis of 6486 patients included in 21 studies, in which the prevalence ranged from 3.5–34% [9]. In most studies, the prevalence of headache in patients with COVID-19 is around 12% [10]. Little information is known about the characteristics of these headaches.

During the pandemic, we noticed frequent clinical visits to our headache clinic with complaints of worsening of previous headache or new onset Headache following COVID-19.

This study aimed to investigate the frequencies, features, and course of pre-existing primary headaches (migraine and tension type headache) as well as the characteristics of the de-novo headache in participants with RT-PCR-confirmed COVID-19.

## Methods

### Study design

This is a cross-sectional study was done at Ibn Sina Hospital, Kuwait. Patients were recruited from headache clinic in Ibn Sina Hospital, which is the only specialized neurology and tertiary hospital in Kuwait. We always encourage our patients to uses headache diary and they bring their migraine diaries papers with them every visit to headache clinic.

### Eligibility criteria

Adult patients aged 18–65 years of both gender who were reviewed in the headache clinic within 3 months after the onset of their COVID-19. Infection with COVID-19 was confirmed by reverse transcription polymerase chain reaction (RT-PCR) technique from material collected by nasal and oropharynx swab. Patients with comorbid medical or mental disorders, or unwilling to participate in the study were excluded.

### Data collection

Neurologists’ experts in headache diagnosis collected data 3 months after COVID-19 infections. They’ve made a detailed descriptive analysis of COVID-19 related headache by face-to-face interviews. A stepwise approach was applied to see if there was pre-existing primary headache disorder according to the ICHD-3 criteria [6]. COVID-19 related headache analysis data included frequency, severity according to the visual analogue scale (VAS) [11], and analgesics use.

Data were collected with a questionnaire. It was reviewed by two independent neurologists and tested on 10 migraine patients for validation. The questionnaire was designed to report demographic and clinical data, including age, gender, attack frequency (times/month), and attack duration (hours), number of analgesics days use/ month and their scores on the visual analogue scale (VAS) [11]. The severity of headache attack was measured by visual analogue scale (VAS) from no pain (=0) to worst pain imaginable = 10. Information regarding the severity of the COVID-19 symptoms. Mild infection defined as quarantine at home, moderate means patient needed admission at hospital and severe infection defined as admission at intensive care.

### Classification of headache disorders

A stepwise approach was applied to see if there was pre-existing primary headache disorder according to the ICHD-3 criteria [6]. Previous headaches presented by these patients were classified according to the third edition of the International Classification of Headache Disorders (ICHD-3). Patients were asked whether they considered the headache after COVID-19 to be similar or different to previous headaches. De novo headache was defined as new onset headache during or immediately after COVID-19 with no prior history of migraine. Characteristics of headache presented post COVID-19 were collected.

### Two co-primary outcomes

First: impact of COVID-19 on primary headache severity, frequency and analgesic use.

Second: characteristics of the de novo headache post COVID-19.

### Ethical considerations

The ethical committee of the Ministry of Health in Kuwait approved the study. Participants were given a simple explanation about the aim of the study. Written informed consent was obtained from all patients before inclusion. Patients were granted the right to decline participation at any time during data collection. All data were protected in accordance with the ethical guidelines of the Council for International Organizations of Medical Sciences and the principles in the Declaration of Helsinki [12, 13].

### Statistical analysis

The statistical analyses were performed using SPSS Statistics Software version 26.0 (IBM Corporation, Armonk, NY, USA). Descriptive data are shown as number (percentage) or mean ± standard deviation for continuous variables, whereas categorical ones were expressed as proportions and percentages. Paired sample *t*-test was used to compare between continuous variables and the chi-squared test(*X*^2^) was used to compare between categorical variables. A *P* value < 0.05 was considered statistically significant.

## Results

A total of 121 patients with headache after recovery from COVID-19 were reviewed at the headache clinic in Ibn Sina hospital, Kuwait was included in this study. Age range was (18–60) years and mean age was 35.29 + 9.54 years. Most of the cohort were females 101 (83.5%).

### Clinical and demographic data

Table 1 presents the Clinical and demographic characteristics of the cohort. Eighty-seven patients aged 40 years or younger. 89 (73.6%) reported primary headache disorders prior to COVID-19, 78 (64.5%) experienced migraine and 11 (9.1%) diagnosed as tension-type headache. 32 (26.4%) reported de novo headache post COVID-19.

**Table 1:** Characteristics of headaches patients with COVID-19 infections Number = 121

Variables	Mean **+** SD/Number (%)
**Mean Age**	35.29 + 9.54
**Range**	18–60
• ≤40 years	87 (71.9)
• > 40 years	34 (28.1%)
**Gender**	
• Male	20 (16.5)
• Female	101 (83.5)
**Job**	
• Full tome	46 (38)
• Part time	24 (19.8)
• Student	13 (10.7)
• Retired	9 (7.4)
• Jobless	29 (24)
**Social state**	
• Single	39 (32.2)
• Married	73 (60.3)
• Divorced	8 (6.6)
• Widow	1 (0.8)
**Severity of COVID infections**	
• Mild	107 (88.4)
• Moderate	14 (11.6)
• Severe	0
**Primary headache before COVID-19 infections**	
• Migraine	78 (64.5)
• TTH	11 (9.1)
• No Headache	32 (26.4)
**Change headache character of headache after COVID-19**	
Yes	49 (55.1)
**De novo Headache after COVID-19 infections (*n*** **= 32)**	
Migraine	20 (62.5)
TTH	12 (37.5)

*COVID-19* Coronavirus disease-19, *SD* standard deviation, *TTH* tension type headache

### The course of the pre-existing primary headache after COVID-19

Of the 121 patients, 89 (73.6%) reported primary headache disorders prior to COVID-19 infections (11 experienced a tension-type headache and experienced 78 migraine).

**Fig. 1:**
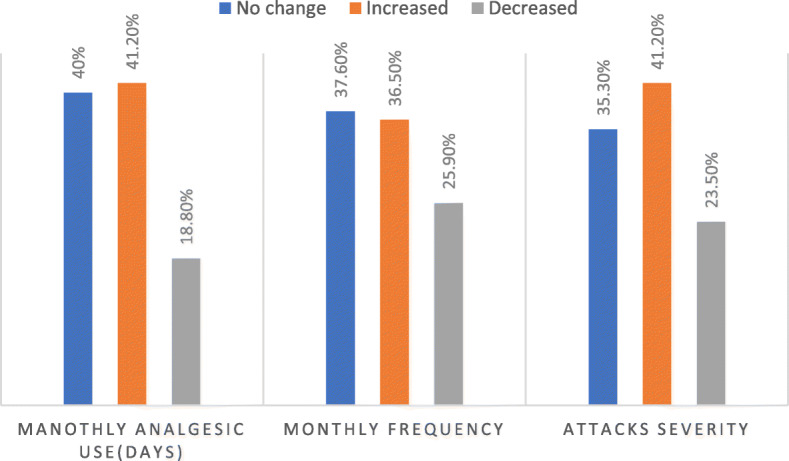
The course of Migraine after COVID-19

Three months after the COVID-19, 41.2% of the patients with migraine reported increase in the attack severity and similar percentage reported increased average days of analgesics use per month and 36.5% reported increased in monthly headache frequency Fig. 1.

**Fig. 2:**
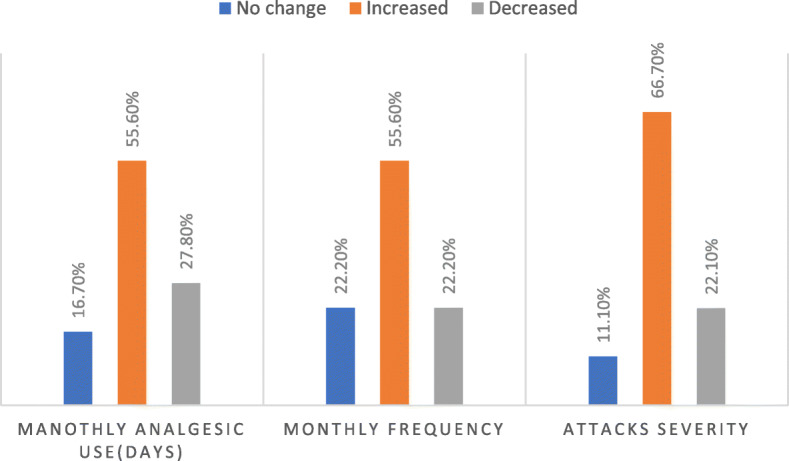
The course of tension type headache post-COVID-19

In the same period, 66.7% of the patients with tension type headache, reported increase in the attack severity and 55.6% reported increase in the monthly attack frequency and similar percentage reported increase in the average days of analgesics use per month. Fig. 2.

Post-COVID-19, the usage of analgesics increased significantly by the patient with migraine (2.31 ± 1.65 vs. 3.05 ± 2.09, *p* = 0.002) while patients with tension type headache had statistically significant increase in severity (5.556 ± 1.86 vs. 7 ± 2.25, *p* = 0.033) and frequency (7 ± 6.29 vs. 12.72 ± 7.96, *p* = 0.006) of headache attacks, Table 2.

**Table 2:** Impact of COVID-19 of preexisting headache

Variables	Patient with Migraine		***p***	Patients with TTH		***p***
	Before infection	After infection		Before infection	After infection	
Attack severity						
Mean ± SD	7.235 ± 2.05	7.42 ± 2.36	0.501	5.556 ± 1.86	7 ± 2.25	0.033*
Headache days per month						
Means± SD	8.18 ± 7.36	8.55 ± 7.17	0.584	7 ± 6.29	12.72 ± 7.96	0.006*
Analgesic use days per month						
Means± SD	2.31 ± 1.65	3.05 ± 2.09	0.002*	2.28 ± 1.74	3.06 ± 2.44	0.177

*COVID-19* Coronavirus disease-19, *SD* standard deviation, *TTH* tension type headache

*=*P* < 0.05, Paired sample t-test

### Changes in characteristics of headache post COVID-19 infection

Forty-nine (55.1%) of these patients reported that the current headache as being different from previous headaches.

Post COVID-19, the patient had significant increase in headache days 11.09 ± 8.45 compared with 8.66 ± 7.49 headache days before COVID infection (*p* < 0.006). Also, days of analgesic use (8.93 ± 7.11 versus 5.62 ± 4.78; *P* < 0,001) and headache severity (8.06 ± 1.70 versus 7.20 ± 1.92; *P* < 0,001) were significantly increased, Table 3.

**Table 3:** Change of headache characters of after COVID-19

Variable	Characters of headache before COVID-19	Characters of headache after COVID-19	***P*** value
**Mean Severity of headache**	7.20 + 1.92	8.06 + 1.70	0.001*
**Mean Frequency of headache/month**	8.66 + 7.49	11.09+ 8.45	0.006*
**Mean Number of analgesics days/months**	5.62 + 4.78	8.93+ 7.11	0.001*

*=*P* < 0.05, Paired sample t-test, *COVID-19* Coronavirus disease-19

The severity of migraine post-COVID-19 significantly increased among female patients. Patients with migraine who had more frequent headache attacks before infection, had significantly increased headache frequency and analgesics use after the infection. Furthermore, patients with more severe attacks before the infection had significantly increased attack severity post-infection. For the patients with tension type headache, while the younger patients had significantly increase in attack severity, female patients had significantly increase in analgesic use post-infection Table 4.

**Table 4:** Characteristics of Post COVID-19 headache changes in relation to age, gender COVID-19 severity and baseline Headache frequency and severity

Variables	Migraine (post- infection)				TTH (post- infection)			
	N/% (*N* = 78)	frequency	Analgesic use	severity	N/% (*N* = 11)	frequency	Analgesic use	severity
Age range:								
• ≤40 years	60/76.9	9.19 ± 7.56	3.03 ± 2.14	7.50 ± 2.34	8/72.7	14.26 ± 7.78	3.27 ± 2.28	7.60 ± 1.39
• > 40 years	18/23.1	7.08 ± 7.17	3.08 ± 1.99	7.24 ± 2.31	3/27.3	5.0 ± 2.64	3.06 ± 2.43	3.66 ± 3.05
***P***		0.224	0.928	0.118		0.063	0.428	0.002*
Gender:								
• Male	11/14.1	6.33 ± 6.97	2.42 ± 2.04	5.91 ± 3.05	2/18.2	11.75 ± 7.67	0	5.5 ± 2.08
• Female	67/85.9	8.91 ± 7.18	3.97 ± 2.60	7.67 ± 2.15	9/81.8	13.0 ± 8.30	3.93 ± 2.01	7.49 ± 2.17
***P***		0.250	0.078	0.016*		0.791	0.002*	0.134
COVID-19 infection severity:								
• Mild	68/87.2	8.77 ± 7.38	3.01 ± 2.06	7.50 ± 2.28	10/90.9	12.58 ± 8.18	3.24 ± 2.38	6.94 ± 2.30
• Moderate	10/12.8	7.09 ± 5.57	3.27 ± 2.37	6.90 ± 2.91	1/9.1	15.0 ± 0	0	8.0 ± 0
***P***		0.472	0.511	0.771		0.778	0.206	0.661
Pre-infection headache frequency								
• < 5 days	38/48.7	5.22 ± 4.79	2.48 ± 1.98	7.40 ± 2.35	5/45.5	12.75 ± 8.15	2.63 ± 2.56	7.5 ± 1.6
• ≥5 days	40/51.2	11.51 ± 7.67	3.56 ± 2.07	7.44 ± 2.36	6/54.5	12.7 ± 7.20	3.4 ± 2.41	6.6 ± 2.67
***P***		< 0.0001*	0.017*	0.935		0.990	0.519	0.415
Pre-infection headache severity								
• Mild	8/10.3	5.55 ± 6.12	3.11 ± 2.57	5.88 ± 3.17	2/18.2	10.0 ± 3.55	2.5 ± 2.88	5.75 ± 3.59
• Moderate	30/38.5	10.03 ± 8.03	3.26 ± 2.16	7.0 ± 2.19	8/72.7	12.45 ± 7.36	3.0 ± 2.32	7.18 ± 1.72
• Severe	40/51.2	8.13 ± 6.62	2.89 ± 1.98	8.02 ± 2.13	1/9.1	17.33 ± 14.18	4.0 ± 3.0	8.0 ± 2.0
***P***		0.22	0.752	0.001*		0.503	0.742	0.411

*=*P* < 0.05 Paired sample t-test, *COVID-19* Coronavirus disease-19, *N* number, *TTH* tension type headache

### Characteristic of the de novo headache

Most of patients who got developed headache post COVID-19 had migraine phenotype (62.5%).

**Fig. 3:**
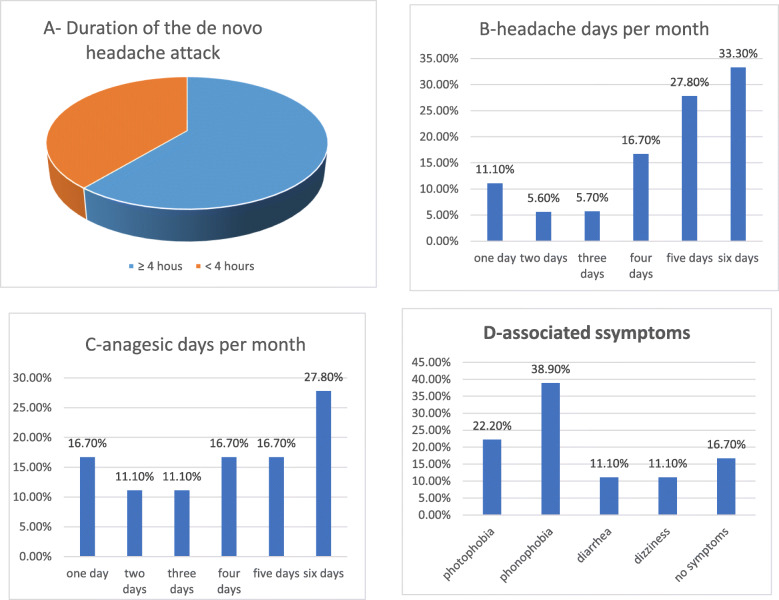
The characteristics of de novo headache; (**A**) Attack duration (**B**) Headache days per month, **C** days of analgesic use per month,(**D**) associated symptome of the headache attack

All the patient with de novo headache had their headache onset during the covid-19 infection. Bi-frontal and temporal headache were the most frequently reported (40.6% each). Half of the patients reported throbbing headache and one third had pressing headache. Mean headache duration was37.27 ± 05.37 days post COVID-19 infections. Headache resolved in most of them 56.3% within 1 month after COVID-19. Moderate headache was frequently reported in 40.6% followed with severe headache in 34.4%. Mean headache attack duration was 14.17 ± 15.46 h. Headache days per month ranged between 1 day (11.1%) and 6 days (33.3%) with means 4.44 ± 1.68. Days of analgesic use per month ranged between 1 day (16.7%) and 6 days (27.8%) with means 3.56 ± 2.85. Phonophobia (38.9%) and photophobia (22.2%) are the most reported associated symptoms Table 5 and Fig. 3.

**Table 5:** Characteristic of the de novo Headache (*N* = 32)

Variable	N (%)
Site:	
• Diffuse	3 (9.3)
• Temporal	13 (40.6)
• Bi-frontal	13 (40.6)
• Occipital	3 (9.3)
Character:	
• Throbbing	16 (50)
• Pressing	11 (34.4)
• Exploding	3 (9.4)
• Dull aching	2 (6.2)
Duration of headache post COVID-19 infection:	
• < One month	18 (56.3)
• One month - < Three month	5 (15.2)
• >Three month	9 (28.1)
• Mean ± SD in days	37.28 ± 5.37
Mean ± SD duration of attacks in hours.	14.17 ± 15.46
Severity:	
• Mild	8 (25)
• Moderate	13 (40.6)
• Severe	11 (34.4)

*COVID-19* Coronavirus disease-19, *SD* standard deviation, *N* number

Younger patients had longer duration of headache attack (18.50 ± 16.44 vs 5.5 ± 9.07, *p* = 0.045). Male patients compared to females (8.66 ± 1.15 versus 5.93 ± 2.01 p = 0.04) had more severe headache. Those who had moderate COVID-19 infection had more severe headache attacks compared to mild COVID-19 infection COVID-19 infection, (6.12 ± 2.06 versus 8.50 ± 2.12, *p* = 0.044) Table 6. We did not report severe COVID-19 infections.

**Table 6:** Characteristics of the de novo headache post- COVID-19 infection in relation to age, gender and severity of COVID-19 infection

Variables	Frequency of de novo headache/ Month (means ± SD)	Attack severity of de novo headache (VAS) (means ± SD)	Attack duration of the de novo headache (hours) (means ± SD)
Age range			
• ≤40 years(*n* = 24)	4.17 ± 1.94	6.16 ± 2.20	18.50 ± 16.44
• > 40 years(*n* = 12)	5.0 ± 0.894	6.83 ± 2.03	5.5 ± 9.07
*P*	0.339	0.196	0.045*
Gender			
• Male (*n* = 6)	6.0 ± 0.0	8.66 ± 1.15	24.66 ± 23.60
• Female(*n* = 26)	4.13 ± 1.69	5.93 ± 2.01	12.06 ± 13.64
*P*	0.079	0.04*	0.207
Covid-19 infection severity			
• Mild (*n* = 25)	4.25 ± 1.69	6.12 ± 2.06	14.15 ± 15.95
• Moderate (*n* = 7)	6.1 ± 0.0	8.50 ± 2.12	13.0 ± 15.55
*P*	0.174	0.044*	0.093

*=*P* < 0.05 Paired sample t-test, *COVID-19* Coronavirus disease-19

## Discussion

COVID-19 is a systemic inflammation affecting all age groups, with high mortality rate and severe adverse outcomes. It involves nervous system, blood vessels, lung, heart, liver, gastrointestinal system, kidney, eyes, and other organs [14].

The present study showed that COVID-19 has a significant negative impact on patients with pre-existing primary headache disorder either migraine or tension type headache. De novo primary headache is frequent post COVID-19. Occurrence of headache during the symptomatic phase of COVID-19 can be consider as headache attributed to systemic viral infection [6].

The de novo headache post COVID-19 has migraineurs features like i.e., throbbing in nature with associated symptoms like photophobia and phonophobia. Migraine features of de novo headache post COVID-19 could be hypothesised that there is a meningeal peripheral senstisation and an activation of the trigemino- vascular system underlying this headache type [15].

Within 3 months after the COVID-19, significant number of patients in this cohort with primary headache had worsening of their headache in form of increased headache attack severity and/ or frequency with subsequent increased analgesics use. Stress during COVID-19 could be a trigger of migraine attacks and may have a role in this worsening. In the other hand worsening of primary headaches could be explained by viral diseases [16].

Long COVID was defined in previous study as the set of symptoms that accompanies the patient even for months after recovery from COVID-19. These symptoms include persistent headache, y fatigue, moderate breathlessness, foggy head, and psychiatric disorders [17]. In our cohort, headache resolved in most of patients within 1 month after COVID-19 and in resolved in the others after 3 months. Persistent headache for at least 6 months, both as a new onset or worsening/ chronicization of a pre-existing migraine should not be underestimated [18].

Post-COVID-19, while the increase in migraine attack severity was noticed more among females and those who had more severe headache attack before COVID-19, the increase in headache attack frequency and analgesic use were more among patients with migraine who had more frequent migraine attacks before infection. For the patients with tension type headache, while the younger patients had significantly increased attack severity, the female patients had significantly increase in analgesics use post-infection. Our results are consistent with those of Magdy et al. [19] who found that patients with pre-existing primary headache disorder had significantly more frequent headache attacks post- COVID-19 infection. On the other hand, in the study by Uygun et al. [20] about headache characteristics in COVID-19 pandemic, which included a total of 3458 participants and 262 participants had confirmed COVID-19 diagnosis, the majority of patients did not report a worsening of previous primary headaches. The better outcomes in Uygun et al. cohort could be explained by avoiding stressful social interactions, consumption of a healthy diet, practicing mild sports activities and reducing the stress of daily work life during the pandemic among his study population.

Previous Studies found that pre-existing primary headache disorders are usually associated with atypical pain process [21] due to atypical release of pro-inflammatory cytokines and chemokines [22], such changes lead to sensitization of central and peripheral nociceptive pathways with a subsequent reduction in pain threshold [23]. That might explain the increased intensity of headache in those with primary headache disorders in our study population.

It is important to keep in mind that as the COVID-19 pandemic rapidly sweeps across the world, it is inducing a considerable degree of negative economic and psychosocial consequences that may contribute to poor mental health. COVID-19 related headache was a commonly reported symptom in many studies, but there was a great diversity in its frequency, severity, character, and duration [24]. The prevalence of headache was ranged from 3.5–34% in previous studies [9]. We reported here a de novo post-COVID-19 headache in 26.4% of patients; their headache started during the active phase of SARS-COV2 Infection. Headache resolved in 56.3% within 1 month while 28.1% have headache for more than 3 months. The headache characters were of migraine phenotype Analgesics use for three or more days was noted among 72.2% of the patients. Longer attack duration was noted among younger patients ≤40 years, while the higher attack severity was noted among males and the those who had more severe COVID-19. Our results are similar to that of Bolay et al. [10] who found that approximately 6%–10% of symptomatic COVID-19 patients reported new-onset, moderate-severe, bilateral headache with pulsating or pressing quality in the temporo-parietal, forehead, or periorbital, of sudden to gradual onset, poorly responsive to common analgesics, with high relapse rate, that was limited to the active phase of the COVID-19. The headache was worse among males compared to females in our cohort. This result is in agreement with Uygun et al. study [20].

Headache can be the prodromal symptom of COVID-19 which could be predictive of a shorter COVID-19 clinical course [25]. On the other hand, disabling headache can persist after COVID-19 resolution. Late-onset headache ascribed to high cytokine levels.

In a follow-up study by Caronna et al. 37.8% (28/74) had ongoing headache after 6 weeks, of those, 50% (14/28) had de novo post-COVID-19 headache [25]. Headache was the prodromal symptom of COVID-19 in 21.9% (7/32) of patients. A total of 62.5% of patients (30/32) had daily constant and poorly responsive to headache treatment. The recent study by Magdy et al. [14] has found that most of the patients had headache onset during COVID-19 (57%) with diffuse headache (52.9%), pressing character (40.7%) with a median intensity of 7, a median duration of 6 h, and median frequency of 7 days/week [19]. In another study by Caronna et al., of 130 patients, 74.6% (97/130) had headache with COVID-19 infection [25].

The neurological symptoms post-COVID-19 may be explained by different pathophysiological bases as direct neuro-invasion with a damage on the neuronal pathway, indirect effects mediated by hypoxia, hypertension, coagulopathy and cytokine storm on the CNS, up to the worsening of pre-existing brain diseases or new disorder [26]. Previous reports showed that COVID-19 has neuroinvasive potential via various pathways [27]. The angiotensin-converting enzyme 2 (ACE-2) receptor, through which COVID-19 appears to cause infection, is primarily present in epithelium of the lungs; however it is also found in the brain, particularly the brainstem [28]. It has been hypothesized that SARS-CoV-2 can invade peripheral trigeminal nerve terminals and enter the central nervous system via transsynaptic pathways [27, 29] and/ or invade meningeal endothelial cells, which are characterized by high angiotensin-converting enzyme 2 receptor expression [30]. Another mechanism of COVID-19 viral entry into the brain may be through the olfactory bulb via trans-synaptic route [28].

In addition, headaches attributed to systemic infection might be caused by the production of pro-inflammatory and nociceptive mediators such as interlukin -1beta, interliukin-6, tumour necrosis factor-alpha, nitric oxide and prostaglandins [31]. Activation of the trigeminovascular system by inflammation or direct involvement of SARS-CoV-2 could explain the migraine like nature of COVID-19 related headache.

Considering these facts, when seeing patients presenting with these characters of headache, physicians should consider SARS-CoV-2 infection as a differential diagnosis to avoid delayed diagnosis or misdiagnosis and prevention of transmission. Physicians should pay more attention to neurological symptoms of COVID-19 in order to avoid chronicization.

Physicians should consider headache during the course of COVID-19 as a possible prognostic factor for the severity of symptoms and for the possible development or worsening of neurological symptomsor headache as sequelae of COVID-19. Headache should be taken into consideration as a possible chronic sequela of the COVID-19, despite it does not be proved as a prognostic factor of COVID-19 severity [26]. Headache also could be considered a prognostic factor for COVID-19 positive evolution and its severity [32]. A recent concept is raised that the international scientific community should use headache as prognostic factor of COVID-19 duration or severity in a COVID-19 clinical setting. Such concept is expressed in two studies, Caronna and Magdy [19, 25].

## Conclusion

The COVID-19 pandemic has a characteristic effect on the course of headaches in individuals with and without pre-existing primary headache disorders. We concluded that post-COVID-19 headaches are significantly more intense and frequent with the migraine-type being the most common. For an accurate diagnosis and disease spread prevention.

Worsening of COVID-19 related headache was higher in patients with primary headache group disorders. Meanwhile, high pain intensity was associated with male gender, younger age and moderate COVID-19 infection. Headache post COVID-19 improved within 3 months. We can reassure our patients that headache will get back to normal after few months post COVID-19.

### Recommendation

Long-COVID headache is a new entity to be closely flooded in these patients. Further studies are needed for better better understanding impact of COVID-19 on headache in order to provide better care and optimal management plan for those patients. Larger studies evaluating the experience of COVID-19 in patients with a history of a primary headache disorder and to assess the time of improvement of their headache post COVID-19 infection.

### Limitations of the study

The limitation of our study are short follow-up period and relatively small size of cohort. Despite we used headache diary, still there is recall biased.

### Strength of the study

Our strength is that the headache-specialist neurologists were directly involved in data collection, which made detailed headache history and characteristics more reliable. To our knowledge, we report the first experience with COVID-19 related headache in Arabian Gulf. This study represents comprehensive description of COVID-19 related headache.

## Data Availability

Authors report that the datasets used for the current study are available from the corresponding author on request.
